# P07 A Phase 3 randomized, double-blind, comparator-controlled study to evaluate safety, tolerability and immunogenicity of V114 pneumococcal vaccine in haematopoietic cell transplant recipients (PNEU-STEM)

**DOI:** 10.1093/jacamr/dlac133.011

**Published:** 2023-01-19

**Authors:** Marissa Wilck, Oliver A Cornely, Per Ljungman, Catherine Cordonnier, Juan Diego Velez, Ron Dagan, Peter Richmond, Qiuxu Chen, Caroline Daus, Kateasha Geddie, Tina Sterling, Tulin Shekar, Luwy Musey, Ulrike K Buchwald

**Affiliations:** Merck & Co. Inc., Kenilworth, NJ, USA; University Hospital of Cologne, Cologne, Germany; Karolinska University Hospital and Karolinska Institutet, Stockholm, Sweden; Henri Mondor Hospital, Créteil, France; Fundacion Valle del Lili, Cali, Colombia; Ben-Gurion University, Beer-Sheva, Israel; University of Western Australia, Perth, Australia; Merck & Co. Inc., Kenilworth, NJ, USA; Merck & Co. Inc., Kenilworth, NJ, USA; Merck & Co. Inc., Kenilworth, NJ, USA; Merck & Co. Inc., Kenilworth, NJ, USA; Merck & Co. Inc., Kenilworth, NJ, USA; Merck & Co. Inc., Kenilworth, NJ, USA; Merck & Co. Inc., Kenilworth, NJ, USA

## Abstract

**Background:**

Allogeneic haematopoietic cell transplant (allo-HCT) represents a definitive therapy for a variety of haematological diseases. Immunosuppressive therapy following allo-HCT and graft-versus-host disease (GVHD) render the patient susceptible to infectious complications, including invasive pneumococcal disease. Post-allo-HCT pneumococcal vaccination is recommended 3–6 months after transplant in a multi-dose series to help prevent pneumococcal disease.

**Objectives:**

V114 is a 15-valent pneumococcal conjugate vaccine (PCV) approved in adults, containing all 13 serotypes in Prevnar 13™ (PCV13; contains serotypes 1, 3, 4, 5, 6A, 6B, 7F, 9V, 14, 18C, 19A, 19F, 23F) as well as epidemiologically important serotypes 22F and 33F. This study evaluated safety and immunogenicity of V114 when given to allo-HCT recipients.

**Methods:**

Individuals ≥3 years of age were randomized 1:1 to receive 3 doses of V114 or PCV13 in one-month intervals starting 3–6 months after allo-HCT. At 12 months after HCT, participants received either PNEUMOVAX^TM^ 23 or a fourth dose of PCV if they experienced chronic GVHD. All participants or their legal representatives provided informed consent at enrolment. Primary safety objective was to evaluate the proportion of participants with adverse events (AEs) within each vaccination group for 3 to <18 years old and ≥18 years old age groups. Primary immunogenicity objective was to evaluate serotype-specific immunoglobulin G (IgG) geometric mean concentrations (GMCs) for all 15 V114 serotypes at 30 days following the third PCV dose (Day 90) in each vaccination group. A key secondary objective was to evaluate opsonophagocytic activity (OPA) geometric mean titres (GMTs) for all V114 serotypes at Day 90.

**Results:**

A total of 274 subjects (14 children [3 to <18 years of age] and 260 adults ≥18 years old) were enrolled and vaccinated in the study. The proportion of participants with systemic AEs and serious AEs after any dose of study vaccine were generally comparable between intervention groups in the paediatric and adult cohorts. V114 recipients reported a higher proportion of injection-site AEs and vaccine-related systemic AEs compared with PCV13 in the adult cohort. Of all vaccinated participants, 32.4% experienced serious AEs during the study (28.8% in V114 group, 36.3% in PCV13 group). Seventeen participants (5.8% in V114 group, 6.7% in PCV13 group) died during the study. None of the deaths was determined by the investigator to be related to study vaccines. For both IgG GMCs (Table 1) and OPA GMTs, V114 was generally comparable to PCV13 for the 13 shared serotypes, and higher for serotypes 22F and 33F at Day 90.

**Conclusions:**

V114 was well tolerated in allo-HCT recipients with a generally comparable safety profile to PCV13 for both paediatric and adult participants. Despite the immunocompromised status of the participants, the study vaccines induced serotype-specific immunogenicity and V114 induced generally comparable responses to PCV13 by IgG GMCs and OPA for the 13 shared serotypes at Day 90. In addition, V114 induced higher antibody responses than PCV13 for serotypes unique to V114 (22F and 33F). Study results support the use of V114 in allo-HCT recipients.

**Table 1.** Serotype-specific IgG geometric mean concentrations at Day 90

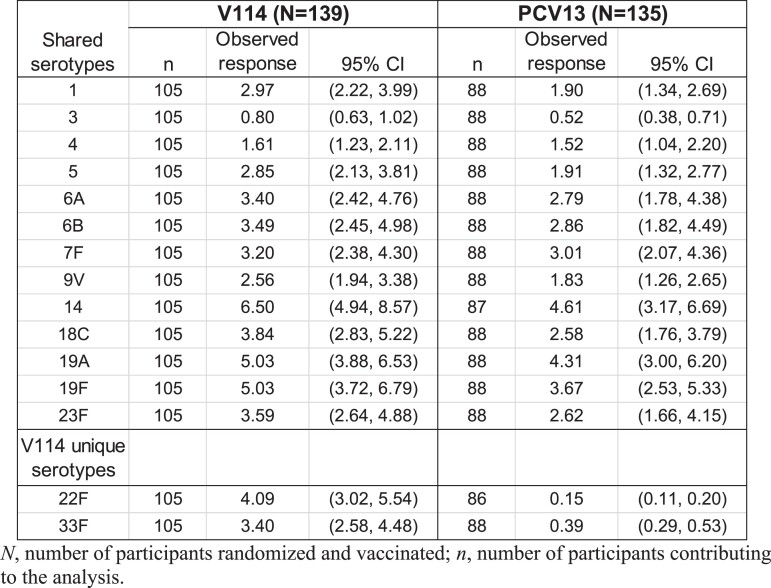

*N*, number of participants randomized and vaccinated; *n*, number of participants contributing to the analysis.

Analysis performed on the per protocol population.

